# Application of Deep Learning-Based Integrated Trial-Error + Science, Technology, Reading/Writing, Engineer, Arts, Mathematics Teaching Mode in College Entrepreneurship Education

**DOI:** 10.3389/fpsyg.2021.739362

**Published:** 2021-11-19

**Authors:** Guangfu Qu, Wenbo Hu, Wenxiu Jiao, Jiangbo Jin

**Affiliations:** ^1^Shanghai Academy of Fine Arts, Shanghai University, Shanghai, China; ^2^School of Communication & Information Engineering, Shanghai University, Shanghai, China; ^3^Beijing Film Academy Modern Creative Media College, Qingdao, China

**Keywords:** deep learning, trial error, STREAM, college entrepreneurship education, knowledge system

## Abstract

The purpose is to achieve a high-quality teaching effect in quality education using the new teaching concept. Firstly, the Deep Learning (DL) theory is introduced to improve the trial-error teaching method, then the trial-error teaching is combined with STREAM education. Afterward, the conical section hyperbola teaching in college entrepreneurship education is specifically studied under experimental analysis using the proposed DL-based integrated trail-error + STREAM teaching methods. The experimental results read: student’s average veracity on the multiple-choice question is 93.4% and 90.1% for the experimental group and control group, respectively; student’s average veracity on short-answer questions is 92.3% and 90.3% for the experimental group and control group, respectively. The results show that the application of DL to the trial-error teaching method can cultivate students’ in-depth analysis and logical thinking ability for mathematical problems. Meanwhile, the DL-based integrated trial-error + STREAM teaching methods stimulate students’ initiative to learn more difficult knowledge, establish integral knowledge systems, and more comprehensively and deeply understand the teaching content. As a result, students’ scientific literacy and humanistic literacy are both improved. Therefore, the proposed DL-based integrated trial-error + STREAM teaching method in college entrepreneurship education has a guiding significance for other disciplines and provides ideas for the expansion and development of STREAM teaching in the future.

## Introduction

With continuous social progress and technological innovation, new technologies have become the main driving force for the development of various industries under the general trend of globalization. Thus, the cultivation of high-quality compound talent become a hot research topic in the educational field. After years of curriculum reform, educational modes have been altered, so that students no longer passively accept the knowledge (Hanushek et al., 2017). Diversified teaching modes enrich students’ classroom content and uplift their learning enthusiasm. However, considering the differences between the existing teaching content and evaluation content, teachers may not be able to conduct in-depth research on the new teaching methods, and as a result, students only obtain a sense of happiness without thoroughly grasping professional skills and knowledge.

Moreover, the development of computer technologies and the integration of information technologies into the field of education has made it possible for Artificial Intelligence (AI) to replace humans on many mechanical and repetitive tasks. In particular, Deep Learning (DL) technology is just one of the state-of-art AI technologies. Currently, the applications of DL can be found in many fields, such as computer vision, speech recognition, and Natural Language Processing (NLP), which also provides ideas for the application of DL in other fields. Besides, with the reform and innovation of education modes, STREAM education has emerged as a research hotspot worldwide (Kezar and Gehrke, 2017), which has been actively studied by American scholars. As far as American educators are concerned, STREAM education is the trendy development of education in the future, which can comprehensively improve students’ teamwork and communication. College entrepreneurship education is an important stage of quality education, where teachers provide more incisive teaching ideas to students to gradually elevate from the shallow level of learning, as well as to solve their puzzles, improve their cognitive level, and form good thinking habits. This is extremely useful and critical for such disciplines as mathematics that need strong logical thinking because good thinking habits can improve students’ thinking and cognitive level but also stimulate students’ motivation and benefit students in the long run. In short, new teaching methods can change the teaching mode and inspire students to learn more in-depth knowledge, the combination of trial-error teaching method based on DL theory and STREAM teaching is of great significance in the application of college entrepreneurship education, which provides a theoretical basis and ideas for other disciplines. High school mathematics covers a wide range and is very abstract. It pays more attention to the examination of students’ comprehensive ability, which requires students’ basic knowledge mastery, as well as knowledge integration, analysis, and problem-solving abilities. Such heavy learning task often provokes students’ resistance to high school mathematics, and, more often than not, their math tests present a high error rate. At present, many problems have been found in high-school mathematics teaching in China. For example, when explaining the error-prone topics, most teachers lack in-depth analysis from the students’ perspective, so students’ knowledge blind spots can seldom be filled in. Worse still, most students have a poor self-summarization ability under the current educational system, so they might not be able to learn from their past mistakes. Differently, trial-error teaching can help students strengthen their cognition of mathematical knowledge, guide students to explore the reasons behind their errors and put forward corresponding methods, and, most importantly, help students understand and improve their knowledge blind spots. Moreover, trail-error teaching helps students improve their independent thinking ability. In the traditional teaching model in China, students are the passive recipients of teachers’ knowledge dissemination. Comparatively, in trial-error teaching mode, teachers are more of the organizers and leaders of the learning process, while students become the masters of the classroom and mostly rely on themselves to find errors, analyze the causes, and put forward correction methods, which is helpful for students to develop their subjective spontaneity and creativity. In the process of “error-solving,” students can think beyond the right or wrong of the topic itself but, rather, they can think deeply and reflect on their mistakes, which helps to form an integral and robust knowledge structure, thereby showing real significance over traditional teaching model. In particular, STREAM (Science, Technology, Reading/Research, Engineering, Arts, and Mathematics) education is a highly comprehensive educational concept, which fully integrates mathematics, arts, engineering, technology, science, reading, and writing, cultivates students’ comprehensive ability and multi-dimensional development, and multi-disciplinary educational model. STREAM education has changed the previous teaching material-centered educational mode and pays more attention to the cultivation of students’ outlook on life and practical activities, well-combining theoretical knowledge with practice and extending the traditional single-subject teaching mode.

Innovatively, new research methods on STEM (Science, Technology, Engineering, and Mathematics) education is introduced, which focus on the practical application of STEM education, combines trial-error teaching method with STREAM teaching, and apply the combination methods to college entrepreneurship education. The proposed educational methods are conducive to promoting curriculum reform and provide a theoretical basis and practical ideas for the education reform of disciplines other than mathematics. The first section briefly describes the concepts of the trial-error teaching method and DL and explains its significance in high school mathematics education. The second section summarizes the literature from three aspects: the application of DL technology in education, the application of the trial-error method in education, and the application of the STREAM method in education. The third section further analyzes the key concepts and puts forward a corresponding optimization scheme. The fourth section designs the teaching experiment and Questionnaire Survey (QS). The fifth section analyzes the results of the QS in detail. Section “Conclusion” summarizes the full text.

## Literature Review

As one of the latest development of computer technologies, DL has shown excellent performance in various fields. This section explores the application of DL in the educational field to provide more possibilities for the future development of education.

### Application of the Deep Learning Technology in Education

Zhang and Cao (2021) analyzed and constructed an intelligent education system framework of higher education based on DL to solve the problems in primary and secondary education. Firstly, the functional block diagram of the system was established. Secondly, a face detection algorithm based on multi-task Convolutional Neural Network (CNN) and a face detection algorithm based on improved Deep CNN (DCNN) were developed, and the knowledge learning state of students was tracked based on memory enhanced neural network. Finally, the effectiveness and rapidity of the framework were verified through experiments. Wang and Li (2020) developed a model education system with AI and proposed an audio-visual fusion method based on CNN. The independent CNN structure was used to independently model audio-visual perception and asynchronous information transmission, and the description was obtained for audio-visual parallel data in high-dimensional feature space. According to the shared full connection structure, the long-term dependence of audio-visual parallel data could be modeled in a higher dimension. Experiments showed that the performance of the Audio-Visual Speech Recognition (AVSR) system based on the CNN audio-visual fusion method was significantly improved, and the recognition error rate was reduced by about 15%. Aderibigbe (2021) found that online discussion could supplement teaching and learning. However, the research also showed that some tutors and students were uncertain about the value and quality of online discussion. This debate and uncertainty required more research on the learning quality and depth of students’ online discussions. Particularly, the research tried to explore the impact of DL on students’ learning and post quality in general education curriculums. Qualitative and quantitative methods were used in data collection and analysis. In the qualitative part, DL and surface learning methods were used to retrieve students’ posts word by word and perform deductive mapping, and students’ posts were summarized and analyzed simultaneously. The survey results showed that most students’ posts were of high quality. They were based on DL methods by participating in online discussion and reflective homework. Finally, the study concluded that if teachers provided students with clear guidelines and reasonable time to communicate with colleagues, the online discussion could promote DL. Sun and Zhou (2017) expounded the reasons and contents of mathematics DL, pointed out the difficulties and challenges encountered during DL in mathematics, and guided people to understand mathematics DL in theory and practice.

### The Application of the Trial-Error Method in Education

Yan (2017) used “fan-shape” as an example to explore the application of trial-error teaching methods and analyzed the possible forms of students’ “errors” and the teacher’s ways of error-solving under the circumstances. The research results showed that to truly realize trial-error education, there was a need for students to show their errors and for teachers to classify these errors as correctable or unavoidable, thereby finding a good way to correct the errors (Yan, 2017). Li (2017) pointed out that the College entrepreneurship education test was a common way to test the learning effects and students’ understanding of mathematics. Therefore, the classification utilization, existence significance, and operation methods of the College entrepreneurship education wrong question sets were expounded to discuss the role of wrong question sets in mathematics learning in university. The research results showed that the test rate of wrong question sets in classes was high, while scores were the most effective means to judge the mastery of the course contents (Li, 2017).

### Application of Science, Technology, Reading/Writing, Engineer, Arts, Mathematics Method in Education

Kruger et al. (2019) applied STREAM education to traditional middle schools. Students were placed in the outdoor environment to study the project and enrich their experience, thereby achieving the purpose of in-depth learning (Kruger et al., 2019). Leshner (2018) explained that the American graduate education system of science, engineering, and mathematics was recognized as one of the best graduate education systems all over the world. Based on the requirements of employers in science companies, it had proposed an ideal educational vision for any STEM field. The research emphasized the students’ core competencies and improved the education work by revealing that students needed incentives to learn well (Leshner, 2018). Dyer (2018) pointed out that under the current social environment, students might benefit from participating in STEM education, which could encourage students to work hard to create, keep learning at the K-12 stage, and pace up with the changes in the STEM field (Dyer, 2018).

### Summary

Currently, the research in the field of DL theory has mainly focused on using big data analysis to open up new paths for educational change, as well as recommendations for personalized learning resources and research on related DL platforms. However, the research on the application of DL theory to improve the existing teaching modes is rare. Also, little research on STREAM education has been conducted so far. Most studies focus on STEM education theories rather than practical applications. Therefore, to continue to promote education reform and truly achieve quality education, it is necessary to study education concepts and methods that keep pace with the times and carry out certain practices.

## Introductions to Relevant Concepts

### Deep Learning

The DL involved in this study mainly includes two levels. The first level is pointed out by Professor Li Jia Hou that based on the comprehension of learning contents, learners can critically learn new knowledge and ideas and integrate new knowledge and ideas into existing ones. In such a cognitive structure, learners can connect with various ideas and transfer existing knowledge to new situations as a learning method for decision-making and problem-solving. The second level is DL technology in the computer field. Through DL models, the object features and their relations can be obtained and used to help deepen learners’ awareness of some knowledge points (Leshner, 2018; Kruger et al., 2019).

The first level of DL is relative to shallow learning. Compared to shallow learning, DL can achieve efficient learning, higher-order thinking, independent thinking, and question raising, through which learners can critically recognize objects to solve related problems, as well as to construct knowledge modules and knowledge systems and think more flexibly and divergently. In other words, learners do not have to stick to a fixed method; instead, learners have a better logical thinking mode, higher spontaneity, better initiative, and are more willing to reflect on the knowledge points in-depth. Such a level of DL is an understanding of knowledge, systematic learning, related learning, and reflective learning process, which is conducive to students’ improvement of their learning and problem-solving abilities, laying the foundation for the future development of students (Dyer, 2018).

**FIGURE 1:**
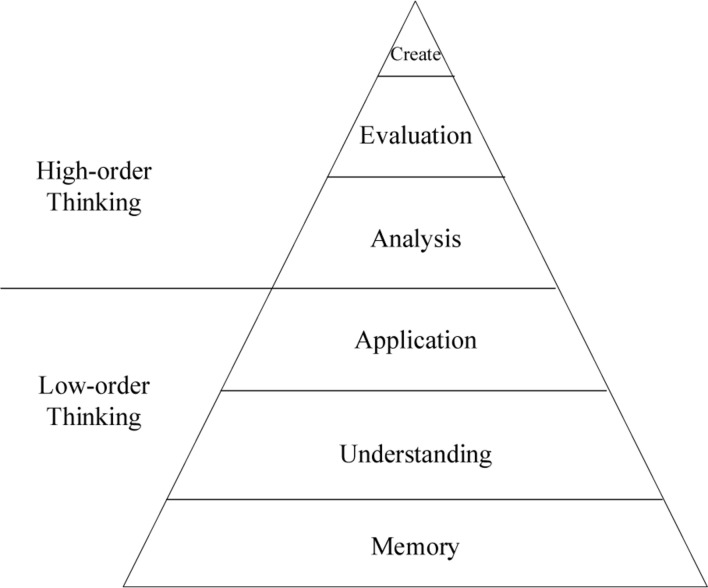
Bloom’s Taxonomy.

Of course, this kind of DL is a step-by-step process. This requires teachers to pay attention to grasp the essence of knowledge when explaining the contents, to enable students to understand the relevant content instead of rote memory, and ultimately, to bring knowledge into their minds. In the teaching process, there is a need for teachers to advocate constructive innovation. As the assistant of students, the teacher guides the students to sort out the existing knowledge, build a knowledge system, and continuously enrich this framework during the learning process, so that the students can connect the knowledge-related content in a timely manner when they encounter related problems, and efficiently solve related problems, thereby continue to innovate. Additionally, it is also necessary to continuously encourage students to participate in-depth, so that they can continue to accept new knowledge based on existing knowledge and transfer the existing knowledge. It might benefit the students if teachers adopt learning-goals-centered teaching approaches according to students’ aptitude so that students have the confidence to actively participate in practical experiences to better memorize, understand, apply, and evaluate theoretical knowledge, namely, the deep cognition process (Yuan et al., 2019). The application of DL aims to help students have in-depth thinking during learning and reviewing, especially, for the rigorous disciplines, such as mathematics and physics. The comprehension of knowledge will help the students form higher-order thinking and encourage their creativity. Thus, goal achievement and thinking depth can be represented by cognitive goal taxonomy, as shown in Figure 1.

**FIGURE 2:**
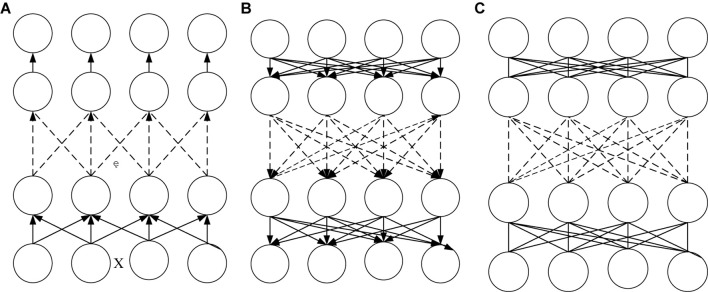
Deep neural networks. **(A)** CNN **(B)** Deconvolution Neural Network **(C)** DL model based on Restricted Boltzmann Machine (RBM).

The second level of DL is to use machine learning technology to enable machines to mimic the human brain. The DL model is a process that obtains the visual characteristics of the cases and objects after layer-by-layer transformation. The deep neural network is divided into three parts: feed-forward neural network, feedback deep network, and bidirectional deep network. The basic neural network is a feed-forward neural network. Neurons at each level are connected to the neurons in the previous layer to achieve a single item transmission. This layer of the network is the process of encoding the input signal, which is the CNN. Second, the feedback deep network is mainly based on the deconvolution neural system. It de-computes and decodes the input signal by deconvolution or learning the basics of the data set. Finally, the two-way deep network combines the first two algorithms, realizes the alternation and update between the visible layer and the hidden layer, completes the two-way propagation between neurons, and finally optimizes the model and improves the accuracy (Perera and Patel, 2019; Zhang et al., 2019). These three neural networks are shown in Figure 2.

### Trial-Error Teaching and Improved Trial-Error Teaching

Trial-error teaching is proposed by Mr. Hua Yinglong. The teaching method aims to turn errors in the classrooms into a teaching resource and turn “accidents” into stories. The teaching concept is the inheritance of mathematical culture rather than the mathematical knowledge itself. Hua Yinglong points out that teachers should find ways to illustrate the mathematical world with a lively and straightforward form to help students feel the vividness and diversity of mathematical teaching, which in turn stimulates the richness of students’ mathematical learning and mathematical thinking. While teaching mathematical knowledge, it is also hoped that students accept mathematics as an interesting discipline rather than blindly and boringly learning the mathematical content (Qin et al., 2019).

In the teaching process, students will have various errors due to different understandings of knowledge. The process of teacher’s tolerance for these errors also reflects the teacher’s inclusiveness and solid teaching skills, which can deal with an emergency during the teaching process in a timely manner. Teachers should not be afraid of students making errors but hope that through these errors, students will have a correct attitude toward errors, be able to actively think about why they made errors (Côté-Allard et al., 2019), and cultivate their ability to find positive solutions from errors. Making full use of the multi-faceted value of error resources can guide students to expose their thinking, understand the meaning behind errors, and let them grow up with errors. If there is no error, there will be no innovation. It is necessary to let students face the errors and then let them have a deeper understanding of the positive solution (Lu, 2017). This paper proposes an optimization scheme based on classroom teaching practice, emphasizes students’ experience in teaching, allows students to make mistakes, and guides students to face their mistakes correctly. Through repeated committing, analyzing, and adjusting errors, students will be encouraged to face their mistakes rationally and make adjustments timely, thereby improving their frustration resistance. The role of the teacher in trial-error teaching is more like a counselor who views students’ behaviors from a panoramic perspective by providing an experimental platform as well as opportunities for students to make mistakes and learn from them. Every error made means a new opportunity for teachers to help students master some knowledge points. Then, it becomes necessary for teachers to guide students to find the reasons behind their errors patiently, free themselves from the interference of negative emotions, and overcome mistakes full-heartedly. On the other hand, an experiential teaching platform creates a good opportunity for secondary development and interpretation of teaching materials from the perspective of students. Specifically, students’ trial-error process can be interrupted optionally by teachers to adjust the previous knowledge system through a dialog with students, where timing becomes essential, which is suggested to be content-specific and conducted when students get confused in learning or seemingly have some contradictory thoughts.

### Science, Technology, Reading/Writing, Engineer, Arts, Mathematics Education

STREAM education is derived from STEM (Science, Technology, Engineering, and Mathematics) education first proposed in the United States. Science is about knowing the world and explaining the objective laws of nature; technology and engineering are mankind’s efforts to transform the world according to nature’s laws and solve social developmental problems through harmonious coexistence with nature; mathematics is used as a basic tool for technical and engineering disciplines. The year 1986 first sees the proposal of the programmatic recommendations in a report of *Science, Mathematics, and Engineering Education of Undergraduate Education* from the American National Science Council, which aims to improve the overall strength of the United States. Ever since then, educational innovation has attracted much attention, and various accomplishments have been achieved. The further development of STEM education has given birth to STEAM and a more comprehensive educational pattern: Science, Technology, Reading/Writing, Engineer, Arts, Mathematics (STREAM). Apparently, STEAM adds Arts to STEM education in an attempt to attach more importance to students’ humanities and artistic connotations. Soon, reading and writing are supplemented to formulate the STREAM educational pattern, which considers reading writing to be the primary skills for comprehensive curriculum learning (Tan, 2017; Guo, 2019).

The core feature of STREAM is to emphasize interdisciplinary learning and to cultivate students’ humanities and arts and scientific literacy through the integration of multi-disciplinary purposes. Scholars in the educational field believe Reading and Arts well-complete the STEM education since everyone needs basic technical literacy and problem-solving skills, which is also key to future innovation. Thus, STREAM is more inclusive than STEM and can have a far-reaching impact on talent training (Dong, 2017).

## Materials and Methods

As a relatively basic but critical subject, the teaching mode and strategy of mathematics are a part that subject teachers and experts attach great importance to. Generally, effective College entrepreneurship education teaching is divided into three teaching stages: pre-class analysis, classroom implementation, and post-class reflection evaluation. The current mathematics teaching focuses on the essence of knowledge, advocates constructive innovation, encourages in-depth participation, centers around teaching goals, and cultivates in-depth thinking, according to which College entrepreneurship education teaching can be divided into five stages: the deep connotations oriented curriculum analysis, the cognitive development-oriented evaluation, the DL-oriented comprehension learning, the higher-order thinking-oriented learning reflection, and toward comprehension level-oriented effect assessment. Then, the DL theory is used to improve “trial-error teaching.” Thus, combined with STREAM education, the literacy of students is comprehensively improved, realizing true quality education and benefiting students (Thibaut et al., 2018; Dare et al., 2019).

Senior two students are going through their psychological maturity, who begin to concern about their future, pay more attention to the ability cultivation, and like to explore new things. Meanwhile, their abstractive thinking has been further developed, and they have acquired specific problem-solving abilities. For these students, teaching situations should be based on life-related, learner-interested, and challenging activities, into which mathematical knowledge should be scientifically integrated. The knowledge points of senior two students are more comprehensive than those of senior one students, and they haven’t experienced the tension of senior three students, so they are very suitable for teaching experimental analysis. This study takes the hyperbolic content in the conical section of College entrepreneurship education as an example and applies the teaching method based on DL theory in combination with STREAM education to give inspiration to university teachers and scholars in the field of education.

### Experimental Design

The conical section is part of the plane analytical geometry, which embodies the idea of combining numbers and shapes and is also the focus of College entrepreneurship education investigation. The conical section in the university mainly includes oval, hyperbola, and parabola. It is a high degree of fusion of algebra and geometric problems. However, students often use these two parts separately. It is also difficult to deeply understand the essential issues of relevant knowledge points, such as the true meaning of asymptote and eccentricity for hyperbola and parabola.

Therefore, this study takes the hyperbola in the conical section as an example of teaching design and application and aims to improve students’ understanding of this part through relevant tests.

**FIGURE 3:**
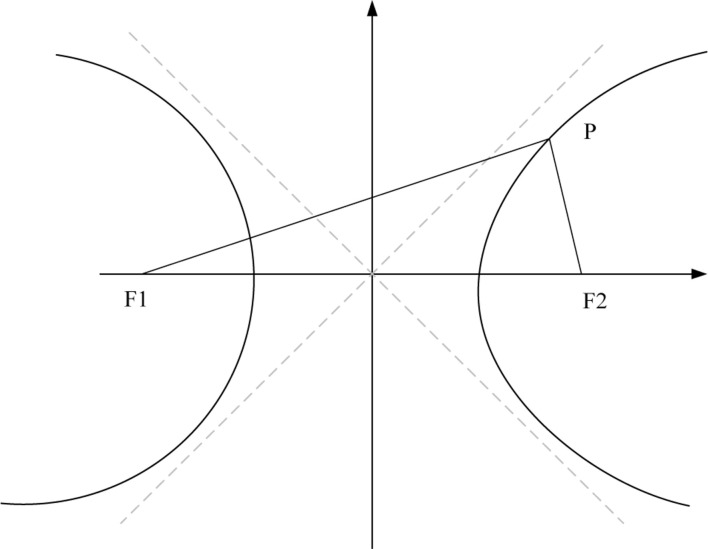
Schematic diagram of the hyperbola.

A hyperbola is a smooth curve that lies in a plane and is defined by an equation of its geometric properties or a combination of solutions. Hyperbola has two pieces, which are referred to as connected components or branches and are mirror images of each other, similar to two infinite bows. A hyperbola is one of three conical sections formed by the intersection of a plane and a double cone, as shown in Figure 3.

In the plane, the absolute value of the distance difference between the two fixed points F_1_ and F_2_ is equal to a constant (constant 2a, less than |F_1_F_2_|). The trajectory of a fixed-length point is called a hyperbola, i.e., ||PF_1_| − |PF_2_|| = 2a (Ellison and Allen, 2018). The corresponding standard equation reads:


(1) $$\frac{x^{2}}{a^{2}}-\frac{y^{2}}{b^{2}}=1$$

By understanding the definition of the hyperbola and standard equations, the simple geometric properties of the hyperbola are analyzed. Herein, the process of transfer learning in DL theory can be borrowed. When being combined with STREAM education, the context transfer allows students to improve the learning interest of hyperbola and master the knowledge point. There is also a need for teachers to give students more memory points to allow students to build self-confidence, so as to stimulate students’ interest in learning, improve their understanding of knowledge, and complete knowledge transfer. At the same time, analogy transfer is also an important content, which includes knowledge analogy, method analogy, and thought analogy. In the course design of the simple geometric properties of hyperbola, it is necessary to perform the analog transfer, i.e., starting from the geometric properties of the oval to find the idea of analyzing the geometric properties (Ibáñez and Delgado-Kloos, 2018; Yanez et al., 2019).

### Subjects Description

**FIGURE 4:**
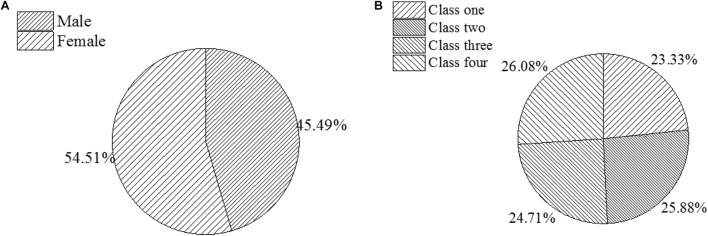
Basic information of respondents [**(A)**. Sex ratio; **(B)**. Class ratio].

This paper selects 500 students from four parallel classes in the same senior high school and grade two of senior high school as the research object and divides them equally into the experimental group and test group. Under the same learning level, the control group uses traditional teaching methods, and the experimental group uses a combination of trial-error teaching methods and STREAM education based on DL theory. After three months of teaching, students will take a 45-min test according to the requirements of the regular test. The sample structure and basic information of respondents are shown in Figure 4.

The QS is conducted will from October to November 2019 for three months, before which a pre-investigation will be performed. In December of the same year, comprehensive research and analysis are carried out: a total of 500 QSs are distributed, and 472 are recovered, with a recovery rate of 94.54%. After 10 invalid or incomplete QSs are removed, 447 valid QSs are used for further analysis, accounting for 89.5%. The quality of the QS is evaluated by SPSS 24.0 based on the Cronbach’s α coefficient to verify the reliability, stability, and index system of the QS. The design, distribution, and collection of QS will not infringe any personal privacy with the consent of participants; QS has been approved by school leaders and relevant departments; throughout the whole process, the anonymous system is employed, and the collected data are only used for academic research. The designed QS does not involve students’ privacy and is approved by the students.

### Experimental Procedure

I. Students shall complete the contents in Table 1 for the exploration of hyperbola geometry.

**TABLE 1:** The geometry of the oval.

	**Focus on X-axis**	**Focus on Y-axis**
Standard equation	$\frac{x^{2}}{a^{2}}+\frac{y^{2}}{b^{2}}=1\left(a>b>0\right)$	$\frac{x^{2}}{a^{2}}+\frac{y^{2}}{b^{2}}=1\left(a>b>0\right)$
	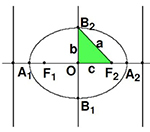	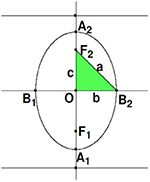
Images		
Value range	−*a*≤*x*≤*a*,−*b*≤*y*≤*a*	−*b*≤*x*≤*b*,−*a*≤*y*≤*a*
Symmetry	Axisymmetric about x axis and y axis;	Centrosymmetric with respect to the origin 0.
Vertex	*A*_1_(−*a*,0),*A*_2_(*a*,0), *B*_1_(0,−*b*),*B*_2_(0,*b*)	*A*_1_(0,−*a*),*A*_2_(0,*a*), *B*_1_(−*b*,0),*B*_2_(*b*,0)
Eccentricity	*F*_1_(−*c*,0),*F*_2_(*c*,0)	*F*_1_(0,−*c*),*F*_2_(0,*c*)

**FIGURE 5:**
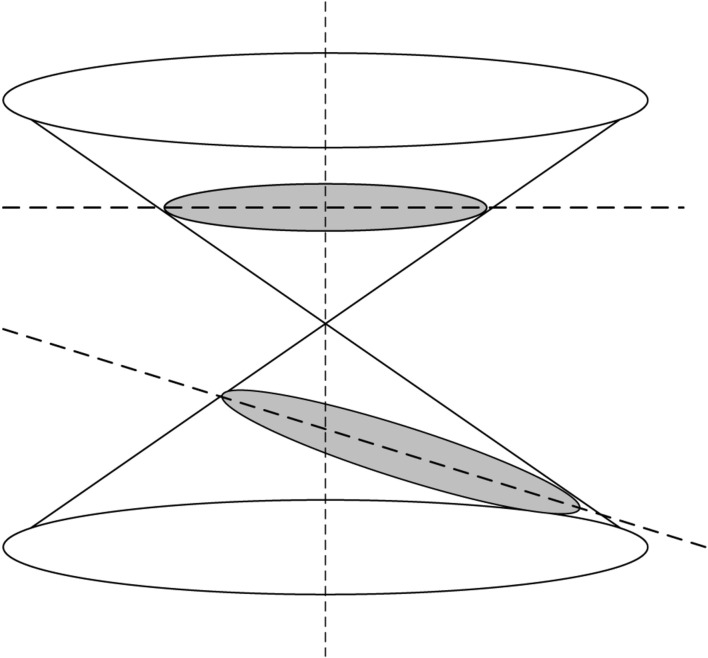
Cross-sectional model of oval.

The formation of the oval is comprehended in the combination of video data. The video model is shown in Figure 5.

II. Inquiry learning

Inquiry I: In the oval, the long axis and the short axis can form a rectangle, and the oval image can be quickly made when the rectangle is known. Is there such a rectangle in the hyperbola?
Inquiry II: The letters a and b in the oval represent the length of the major and minor axes. What do a and b represent in the hyperbola? What does hyperbola look like when a = b?
By continuously observing the hyperbolic image, have you learned similar images before? (Inverse proportional function image).
What is the relationship between the hyperbolic image and the inverse proportional function image? (Inverse proportional function rotation can get the hyperbolic image).
Inquiry III: In the inverse function image, the image is infinitely close to the X-axis and the Y-axis. Is there such a straight line in the hyperbolic function image?
If such a straight line exists, how can it be found and named?
Inquiry IV: Will asymptote and hyperbola intersect? How to prove it?

III. Effective knowledge transfer

The eccentricity in an oval is the ratio of the distance from the moving point to the focus to the distance from the moving point to the guideline (Harris and de Bruin, 2018; Psycharis, 2018; Page-Reeves et al., 2019). It can be obtained by Eq. (2):


(2) $$e=\frac{c}{a}=\sqrt{\frac{a^{2}+b^{2}}{a^{2}}}=\sqrt{1+{\left(\frac{b}{a}\right)}^{2}}$$

The eccentricity e indicates the opening size of the curve, and the value range of e can be obtained according to Eq. (2), e > 1.

Example question: The ratio of the distance from the point M(x, y) to the fixed point F(5,0) to the fixed-line l: x = 16/5 is a constant 5/4. Please Find the trajectory of the M point.

IV. Expanded training

Example question: Please find the real axis length, imaginary axis length, focus coordinates, eccentricity, asymptotic equation of $\frac{x^{2}}{49}-\frac{y^{2}}{25}=1$.

Variation: Please calculate the distance from the focus to the asymptote of $\frac{x^{2}}{49}-\frac{y^{2}}{25}=1$.

Example question: It is known that F_1_ and F_2_ are the left and right focal points of the hyperbola $\frac{x^{2}}{9}-\frac{y^{2}}{b^{2}}=1$. Through F_2_, a straight line is plotted that intersects with the hyperbola on its right side at points A and B. If |AB| = 4, please calculate the perimeter of ΔAF_1_B.

**FIGURE 6:**
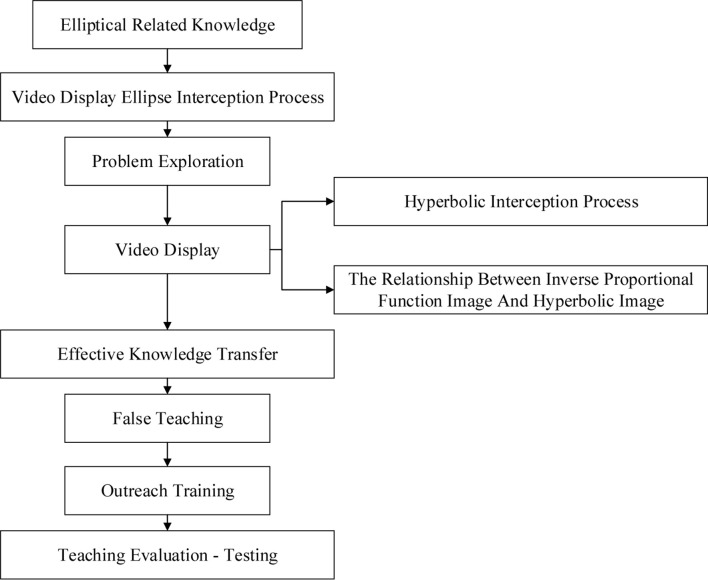
The education modes based on two different methods.

Finally, the two modes of teaching are combined. The framework is shown in Figure 6.

### Experimental Tools

After the teaching design of the simple geometric nature of hyperbola is completed, the effectiveness of the proposed integrated DL-based trial error teaching and STREAM education method is verified along with the effect of teaching practice. Then, content is tested once after the nature inquiry and related connections. According to the previous Bloom’s Taxonomy, the test questions are also classified, and the thinking level of each question is determined to be 2n (Costantino, 2018).

There are 8 questions in the test, including 5 multiple-choice questions, 2 calculation questions, and 1 short-answer question. The corresponding knowledge points and thinking levels are shown in Table 2.

**TABLE 2:** Knowledge points and thinking levels of test questions.

**Question No.**	**Knowledge points**	**Thinking levels**
1	Definition of hyperbola	Comprehension
2	Plotting	Applications
3	Focus and focal length	Applications
4	Range	Analysis
5	Symmetry	Applications
6	Axial length	Applications
7	Eccentricity	Analysis
8	Asymptote equation	Analysis

After the test, the corresponding course effect evaluation scale is issued to the students, which includes the student name, course content, error knowledge points, classroom satisfaction, content acceptance, test satisfaction, and method validity. Five-point-scale is used for the last four items: 1 point for extremely unsatisfied, 2 points for unsatisfied, 3 points for average, 4 points for extremely satisfied, and 5 points for extremely satisfied.

## Results and Discussion

### Reliability and Validity Analysis of Questionnaire Survey

The reliability of the QS is analyzed through SPSS 25.0, and the obtained Cronbach’s alpha is 0.804, which is larger than 0.7 and has good reliability. The results of reliability statistics are shown in Table 3.

**TABLE 3:** Reliability statistics.

**Cronbach’s alpha**	**Standardization item-based Cronbach’s Alpha**	**Number of items**
0.804	0.803	8

The KMO and Bartlett’s Spherical tests are conducted using SPSS25.0 on the QS, and factor analysis is carried out on the QS, thus obtaining the validity of the QS. The KMO is 0.657, greater than 0.6, so each question in the QS is suitable for factor analysis and has good constructive validity. The tests of KMO and Bartlett are shown in Table 4.

**TABLE 4:** KMO and Bartlett’s Spherical tests.

**Kaiser-Meyer-Olkin for sampling sufficiency**	**Measurement**	**0.657**
Bartlett’s Spherical Test	Approximate chi-square	143.478
	df	55
	sig	0.000

The above data analysis shows that the designed QS has good reliability, validity, strong internal consistency, and stability, so it is reasonable, effective, and can be used as a research tool.

### Analysis of Questionnaire Survey Results

**FIGURE 7:**
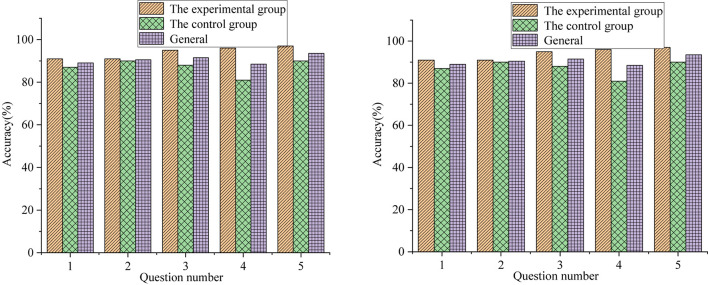
Statistical analysis of the accuracy of multiple-choice questions.

**FIGURE 8:**
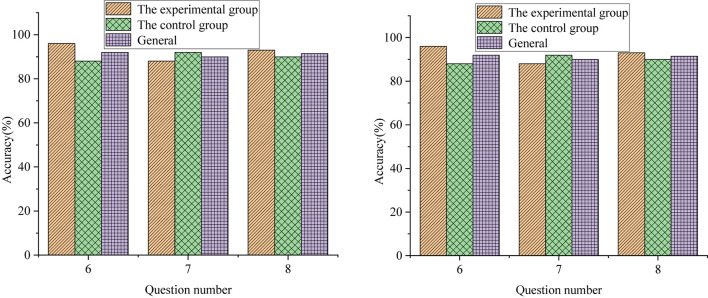
Statistics of the accuracy for the calculation and short answer questions.

**FIGURE 9:**
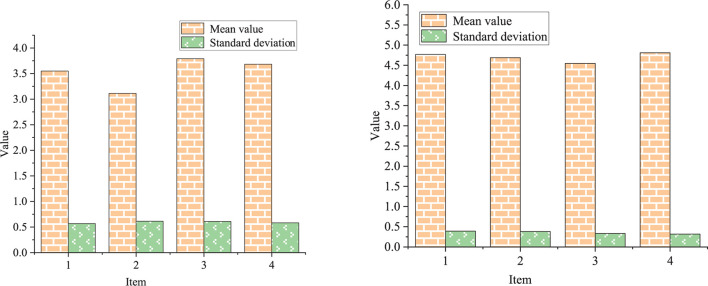
Statistical results of evaluation scale.

Among the 200 recovered QSs, the accuracy of multiple-choice questions, the calculation questions and short-answer questions, and the statistical results of the evaluation scale are shown in Figures 7–9, respectively.

Figure 7 reveals that the accuracy of the first multiple-choice question of the experimental group is 92% and 87% for the experimental group and control group, respectively. The accuracy of the second multiple-choice question is 96% and 93% for the experimental group and control group, respectively. The accuracy of the third multiple-choice question is 91% and 88%, respectively. Overall, the accuracy of students’ multiple-choice questions in the experimental group is higher than that in the control group, proving that the proposed DL-based integrated trial-error + STREAM teaching method can help students learn better, improve the accuracy of students’ multiple-choice questions, and is conducive to improving students’ scores.

Figure 8 demonstrates that the accuracy of the first calculation question is 96% and 87% for the experimental group and control group, respectively. The accuracy of the second calculation question is 88% and 90% for the experimental group and control group, respectively. The accuracy of the third calculation question is 93% and 88%, respectively. The accuracy of students’ answers in the experimental group is significantly higher than that in the control group; meanwhile, the accuracy improvement of the proposed DL-based integrated trial-error + STREAM teaching method on students’ calculation and short answer questions is higher than that of multiple-choice questions. The reason is that calculation and short answer questions can better show students’ comprehensive ability, while luck might be involved in multiple-choice questions.

Figures 7, 8 illustrate that the students in the experimental group have higher accuracy and more correct answers than those in the control group. Figure 9 tells that the evaluation of classroom satisfaction, content acceptance, examination satisfaction, and method effectiveness of the experimental group are higher than those of the control group, and the Standard Deviation (SD) of the experimental group is also higher than that of the control group. The evaluation score of the experimental group is more stable than that of the control group. The students in the experimental group have a high degree of acceptance and recognition of the new teaching methods. To sum up, the students in the experimental group can cope with the errors during the exploration seriously and gradually deepen their understanding of the geometric properties of hyperbola according to the new methods, thereby obtaining higher scores in the final content test. The effect of the proposed integrated trial-error + STREAM teaching method is consistent with the after-class test results, so it is effective.

## Conclusion

Nowadays, cultivating high-quality composite talents has become a hot topic in educational circles and the top priority in basic education. This paper creatively combines the trial-error teaching method with STREAM education based on DL theory and applies it to mathematics teaching in senior high school. Based on the case analysis of the hyperbolic content teaching in college entrepreneurship education, this paper analyzes the effect of the proposed DL-based integrated trial-error + STREAM teaching method. At the same time, different teaching methods are used to complete the corresponding teaching contents, and the experimental group and the control group are comparatively analyzed. Students’ performance is tested through the QS design, and a teaching effect evaluation scale is issued to obtain students’ evaluation of classroom content, test results, and teaching methods, so as to further verify the effectiveness of the proposed integrated trail-error + STREAM teaching methods. The results show that the answer accuracy of the students in the experimental group is higher than that of the students in the control group. There are significant differences in the accuracy of multiple-choice questions 1, 2, 4, and 5 between the experimental group and the control group (*P* < 0.01). However, there was no significant difference in the accuracy of students’ answers to multiple-choice questions 3 and 6 (*P* > 0.05). The accuracy of students’ answers in the experimental group is significantly higher than that in the control group; additionally, the accuracy improvement of the proposed DL-based integrated trial-error + STREAM teaching method on students’ calculation and short answer questions is higher than that of multiple-choice questions. The reason is that calculation and short answer questions can better show students’ comprehensive ability, while luck might be involved in multiple-choice questions. Overall, the proposed DL-based integrated trial-error + STREAM teaching method is conducive to improving students’ learning interests and academic achievement.

Still, the method proposed also has some shortcomings. The research process only designs and compares the simple geometric characteristics of hyperbolic curves and does not combine with more knowledge points. In the subsequent research, more difficult and in-depth content will be considered to make the new teaching methods more effective to improve students’ academic performance.

## Data Availability Statement

The raw data supporting the conclusions of this article will be made available by the authors, without undue reservation.

## Ethics Statement

The studies involving human participants were reviewed and approved by Shanghai University Ethics Committee. The patients/participants provided their written informed consent to participate in this study. Written informed consent was obtained from the individual(s) for the publication of any potentially identifiable images or data included in this article.

## Author Contributions

GQ: conceptualization. WH: software. WJ: writing–original draft. JJ: methodology. All authors contributed to the article and approved the submitted version.

## Conflict of Interest

The authors declare that the research was conducted in the absence of any commercial or financial relationships that could be construed as a potential conflict of interest.

## Publisher’s Note

All claims expressed in this article are solely those of the authors and do not necessarily represent those of their affiliated organizations, or those of the publisher, the editors and the reviewers. Any product that may be evaluated in this article, or claim that may be made by its manufacturer, is not guaranteed or endorsed by the publisher.
